# Analysis of antibiotic resistance phenotypes and molecular variants in antibiotic resistance genes among *Mycoplasma synoviae* isolates collected between 2018 and 2025

**DOI:** 10.3389/fvets.2026.1884930

**Published:** 2026-07-09

**Authors:** Huaiying Xu, Junfeng Lv, Qiaoya Zhao, Dan Yin, Feng Hu, Xiuli Ma, Xinhua Zhao, Zhuoming Qin

**Affiliations:** 1 Poultry Institute, Shandong Academy of Agricultural Sciences, Jinan, China; 2 Shandong Provincial Key Laboratory of Livestock and Poultry Breeding, Jinan, China

**Keywords:** *Mycoplasma synoviae*, antibiotic resistance phenotypes, minimum inhibitory concentration, resistance gene, molecular variants

## Abstract

**Introduction:**

Antibiotic treatment represents one of the available control strategies for managing clinical outbreaks in flocks infected with *Mycoplasma synoviae* (MS). Therefore, elucidating the antibiotic susceptibility profiles of MS isolates and monitoring the evolutionary trends of resistance-associated genetic mutations are of critical importance.

**Methods:**

In present study, the minimum inhibitory concentrations (MICs) of 12 commonly used antimicrobial agents were evaluated for 35 MS isolates collected between 2018 and 2025 using the broth microdilution method. In addition, the molecular variation characteristics of the quinolone resistance-determining region (QRDR) and macrolide resistance-associated genes were analyzed in the same 35 MS strains.

**Results:**

All MS isolates exhibited low minimum inhibitory MIC values for tylvalosin (MIC₉₀ = 0.25 μg/mL) and valnemulin (MIC₉₀ = 0.0078 μg/mL), indicating high *in vitro* susceptibility to both agents. In contrast, the majority of isolates showed markedly elevated MICs for ciprofloxacin (0.25–32 μg/mL), enrofloxacin (0.5–16 μg/mL), and tilmicosin (0.125–8 μg/mL), suggesting reduced susceptibility or emerging resistance to these antimicrobials. The MIC values for the other ten drugs remained relatively low, indicating preserved efficacy. Two amino acid substitutions Asn345Ser in GyrB and Thr85Ile in ParC led to high level resistance to fluoroquinolones. In domain II of the 23S rRNA gene, nucleotide substitutions G778A and G915C, together with substitution G2761C in domain V, were contributed to the elevated MICs for macrolides, tetracyclines, and cephalosporins.

**Conclusion:**

The results of this study would deepen veterinarians’ understanding of resistance mechanisms and provide scientifically validated data to guide the rational use of existing antimicrobials, as well as to support the development of novel therapeutics against MS infection.

## 1 Introduction

*Mycoplasma synoviae* (MS) is an opportunistic respiratory pathogen capable of inducing subclinical respiratory signs, joint swelling, lameness, weight loss, and eggshell apex abnormalities (EAA) following infection (1, 2). The disease can be transmitted both horizontally and vertically through infected eggs. Moreover, infected poultry often become lifelong carriers, leading to prolonged disease persistence and establishing MS as a significant reservoir pathogen (3, 4).

In the early stage of *Mycoplasma* infection, the use of antibacterial drugs has been proven to reduce clinical symptoms and the occurrence of lesions (5). In production, various antibiotics are used to treat *Mycoplasma* infections for several decades, such as fluoroquinolones, macrolides, and tetracyclines. However, long-term use of antibiotics has also led to antimicrobial resistance (6). Macrolides, lincomycins, and pleuromutilins inhibit protein synthesis by binding to 50S ribosomal proteins, tetracyclines inhibit protein synthesis by binding to 30S ribosomal proteins, and fluoroquinolones inhibit DNA synthesis by binding to DNA translocase enzymes and topoisomerase IV. The antimicrobial resistance of macrolides and fluoroquinolones in mycoplasma is related to point mutations in the 23S rRNA gene and amino acid substitutions in the quinolone resistance determinant regions (QRDRs) of DNA flexibility enzymes and topoisomerase IV genes (7, 8).

In this study, MS strains isolated from clinical cases were collected from 2018 to 2025. The minimum inhibitory concentrations (MICs) of key antimicrobial agents commonly used in poultry practice—including enrofloxacin, tilmicosin, and tylosin—were determined using the microbroth dilution method. Furthermore, resistance-associated genes were sequenced and analyzed to elucidate the molecular mechanisms underlying antimicrobial resistance in MS, thereby providing an evidence-based foundation for improved prevention and control strategies against MS infection in poultry.

## 2 Materials and methods

### 2.1 Strains

Thirty-five MS strains were isolated from large-scale poultry farms in Shandong, Hebei, and Jiangxi provinces between 2018 and 2025. All strains were identified and preserved in our laboratory at −80 °C. During the experiment, the MS strains were recovered and inoculated into modified Frey’s broth medium (pH 7.8), followed by incubation at 37 °C for 24–48 h. MS growth was confirmed when the culture medium exhibited no contamination and its color changed from red to orange or yellow. The cultures were subsequently titrated in modified Frey’s liquid medium to determine the color-changing unit (CCU).

### 2.2 Preparation of antimicrobials solution

The antimicrobial agents used in this study were all purchased from Solarbio Life Sciences (Beijing, China). Each agent was dissolved in the appropriate solvent according to the manufacturer’s instructions. The resulting solutions were diluted with modified Frey’s liquid medium to a concentration of 128 μg/mL and then sterilized using 0.22 μm membrane filters. All stock solutions were stored at 4 °C until further use.

### 2.3 Determination of MIC

The MICs were determined by the broth microdilution method in sterile 96-well cell microtiter plates (9). The MS strain was subcultured in modified Frey’s broth medium, followed by aseptic 10-fold serial dilutions to prepare a standardized inoculum with a final concentration of 10^5^–10^6^ CCU/mL. In the 96-well plates, columns 1–23 received 100 μL of broth medium per well; column 24 served as the reagent blank control and received 200 μL of broth medium (no inoculum or antimicrobial agent). A 100 μL aliquot of the working-concentration antimicrobial solutions were added to each well in column 1, gently mixed using a multichannel pipette, and then 100 μL was transferred to each well in column 2. Two-fold serial dilutions were performed across columns 3–22; after mixing each column thoroughly, 100 μL was removed and discarded from column 22 to maintain uniform final volume. Subsequently, 100 μL of the standardized MS suspension was added to each well in columns 1–23 (excluding column 24), yielding a final assay volume of 200 μL per well. The plates were sealed with an impermeable adhesive film and incubated at 37 °C in a humidified 5% CO₂ atmosphere for 96 h. Growth was assessed visually by monitoring the phenol red–mediated color shift (from red/orange to yellow) indicative of acid production due to mycoplasmal metabolism. The lowest concentration of antimicrobial that prevented any color change was defined as the MIC. Based on the MIC values obtained from multiple isolates, the MIC₅₀ and MIC₉₀ (the minimum concentrations required to inhibit 50 and 90% of the isolates, respectively) were calculated. Susceptibility and resistance were interpreted according to the guidelines of the Clinical and Laboratory Standards Institute (CLSI) and published criteria (9, 10).

### 2.4 Analysis of resistance-determining genes

Genomic DNA of the MS strains were extracted using a Bacterial Genomic DNA Extraction Kit (Tiangen Biotech, Beijing, China). Resistance-related genes, including *gyrA*, *gyrB*, *parC*, *parE*, *rplD*, and *rplV*, were selected, and primers were designed based on previous studies (Table 1). Primers targeting the 23S rRNA gene were designed based on sequences available in GenBank (Table 1). PCR amplification was performed using Phanta Max Master Mix (Vazyme, Nanjing, China), and the resulting PCR products were cloned into the pMD18-T vector (Takara, Beijing, China). Sequencing was carried out by Sangon Biotech Co., Ltd. (Shanghai, China), and the obtained sequences were analyzed using Lasergene 7.1 software and aligned with those of reference MS strains MS-H (GenBank accession number: CP021129) and WVU1853 (GenBank accession number: CP011096). The 23S rRNA gene nucleotide positions were located based on the reference MS strain MS53 (GenBank accession number: NR_076213).

**Table 1:** Primers of PCR reaction of antimicrobial drug-associated resistance genes.

Gene	Primer name	Sequence (5′—3′)	Position
*gyrA*	*gyrA -*F	GAAGATCAGCCTGAATTAGTT	58–78
	*gyrA -*R	GCCATTCTAGCTTCGGTATAA	531–551
*gyrB*	*gyrB -*F	CAAGGTGAGAAATTCTCAAGA	964–984
	*gyrB -*R	TGTGCTTCGTTATAAGCG	1,677–1,694
*parC*	*parC -*F	CCAACCGTGCAATTCCTGAT	95–114
	*parC -*R	TTATGCGGCGGCATTTCG	546–563
*parE*	*parE -*F	GGCATATCGTCGAGGAAATAGC	1,034–1,055
	*parE -*R	AGTGGTTTCCCAAAGTTG	1741–1758
*rplD*	*rplD -*F	GTTAAACCCCAATTTAACG	88–106
	*rplD -*R	GAACCTAGCACCCTTTCTGTA	663–683
*rplV*	*rplV -*F	GGCACAACAAGCAAAAGCAC	3–22
	*rplV -*R	TTATGCACGCTCCTCTAA	325–342
23S rRNA	*rrlA-*F	AGCTATAAATGCGAGACACG	168–187
	*rrlA-*R	ACTTCCATCCTATCAACCTC	2,806–2,825

## 3 Results

### 3.1 MIC determinations

In this study, thirty-five MS isolates were tested for susceptibility to 12 antimicrobial agents (Table 2). The MIC values of the tested agents varied considerably. Among fluoroquinolones, ciprofloxacin (CIP) exhibited an MICs range of 0.25–32 μg/mL, while enrofloxacin (ENR) ranged from 0.5 to 16 μg/mL. For macrolides, the MIC ranges were as follows: tylosin (TYL), 0.0156–4 μg/mL; tylvalosin (TVL), 0.0039–0.5 μg/mL; and tilmicosin (TIL), 0.125–8 μg/mL. Tetracyclines also showed broad activity: chlortetracycline (CTC) MICs ranged from 0.031 to 16 μg/mL and doxycycline (DOX) from 0.062 to 16 μg/mL. The aminoglycoside kanamycin (KAN) had an MIC range of 0.125–32 μg/mL. Pleuromutilins displayed notably low MICs, with valnemulin (VAL) ranging from 0.002 to 0.0156 μg/mL and tiamulin (TIA) from 0.0078 to 4 μg/mL. The lincosamide–aminocyclitol combination lincomycin–spectinomycin (LS) yielded MICs of 0.031–8 μg/mL, whereas the amphenicol florfenicol exhibited an MIC range of 0.5–32 μg/mL (Table 2).

**Table 2:** Minimum inhibitory concentration (MIC) distribution of 12 drugs against 35 MS isolates.

Drugs	Number of isolates within the tested dilution (μg/mL)																Breakpoint (μg/mL)	
	0.002	0.0039	0.0078	0.0156	0.031	0.062	0.125	0.25	0.5	1	2	4	8	16	32	64	Sensitive	Resistant
CIP								1	5	6	8^50^	5	6	3^90^	1		≤1	≥2
ENR									3	4	6	11^50^	5	6^90^			≤1	≥2
TYL				1	4	5	8^50^	7	3	4^90^	2	1					≤1	≥2
TVL		1	3	4	8	7^50^	5	3	4^90^								≤0.5	≥1
TIL							3	5	7	9	4	4^90^	3				≤0.5	≥1
CTC					2	3	6	3	5	3^50^	3	4	3^90^	2			≤2	≥4
DOX						1	2	4	5^50^	4	7	6	3^90^	2			≤2	≥4
KAN							1	3	6	4	3	5^50^	6	5^90^	2		≤4	≥16
VAL	5	24^50^	3^90^	3													≤0.125	≥0.25
TML			4	2	5	8^50^	5	2	2	2	3^90^	2					≤2	≥4
LS					1	2	5	8	9^50^	4	2	3^90^	1				≤2	≥4
FFC									4^50^	3	5	7	9	5^90^	2		≤8	≥16

The gray areas indicate the number of drug-resistant MS strains. Superscript numbers (50 and 90) indicate the MIC_50_ and MIC_90_ values.

The MIC₉₀ values were as follows: CIP, 16 μg/mL; ENR, 16 μg/mL; TYL, 1 μg/mL; TVL, 0.5 μg/mL; TIL, 4 μg/mL; CTC, 8 μg/mL; DOX, 8 μg/mL; KAN, 16 μg/mL; VAL, 0.0078 μg/mL; TIA, 2 μg/mL; LS, 2 μg/mL; and FFN, 16 μg/mL (Table 3). Comparison of these values showed that VAL had the lowest MIC₉₀; TIA, TIL, and TYL exhibited MIC₉₀ values 256-fold, 1,024-fold, and 128-fold higher than that of VAL, respectively (Table 2).

**Table 3:** Molecular characterization of resistance-determining regions of MC isolates.

Strains	MIC(μg/ml)		Amino acid mutations in QRDRs						
	ENR	CIP	GyrA	GyrB			parC	parE	
			63	181	345	417	85	348	446
WVU1853/1955	0.5^a^	/	G	A	N	S	T	V	P
MS-H/1986	0.03^a^	/	E	T	N	S	T	V	P
Weifang-1/2018	0.5	0.25	G	A	N	N	T	V	S
Qingdao-1/2018	1	0.5	G	A	N	N	I	G	S
Weifang-2/2019	2	1	G	A	S	S	I	G	S
Liaocheng-1/2019	8	8	G	A	S	S	I	V	S
Zaozhuang-1/2019	1	1	G	A	N	N	I	V	S
Jinan-1/2019	4	4	G	A	S	S	I	V	S
Dezhou-1/2020	0.5	0.5	G	A	N	N	I	V	S
Weifang-3/2020	8	2	G	A	S	S	I	V	S
Binzhou-1/2020	4	2	G	A	S	S	I	V	S
Dezhou-2/2021	2	1	G	A	S	S	I	V	S
Weifagn-4/2021	2	0.5	G	A	S	S	I	V	S
Liaocheng**-**2/2021	4	2	G	A	S	S	I	V	S
Weifang-5/2021	16	8	G	A	S	S	I	V	S
Jinan-2/2021	4	2	G	A	S	S	I	V	S
Dezhou**-**3/2022	4	2	G	A	S	S	I	V	S
Jinan-3/2022	4	2	G	A	S	S	I	V	S
Weifang-6/2022	1	1	G	A	S	S	I	V	S
Liaocheng**-**3/2022	4	2	G	A	S	S	I	V	S
Weifang-7/2022	4	4	G	A	S	S	I	V	S
Zaozhuang-2/2023	2	1	G	A	S	S	I	V	S
Binzhou-2/2023	8	16	G	A	S	S	I	V	S
Qingdao-2/2023	2	1	G	A	S	S	I	V	S
Jinan-4/2023	1	0.5	G	A	N	N	T	V	S
Binzhou-3/2023	16	32	G	A	S	S	I	V	S
Weifang-8/2023	4	4	G	A	S	S	I	V	S
Linyi-1/2023	0.5	0.5	G	A	N	N	T	V	S
Taian-1/2024	2	4	G	A	S	S	I	V	S
Taian-2/2024	8	4	G	A	S	S	I	V	S
Zaozhuang-3/2024	16	16	G	A	S	S	I	V	S
Yantai-1/2024	4	2	G	A	S	S	I	V	S
Binzhou-4/2024	8	8	G	A	S	S	I	V	S
Linyi-2/2025	16	8	G	A	S	S	I	V	S
Zaozhuang-4/2025	4	8	G	A	S	S	I	V	S
Weifang-9/2025	16	16	G	A	S	S	I	V	S
Liaocheng-4/2025	16	8	G	A	S	S	I	V	S

a Data obtained from previous study (8).

**Figure 1:**
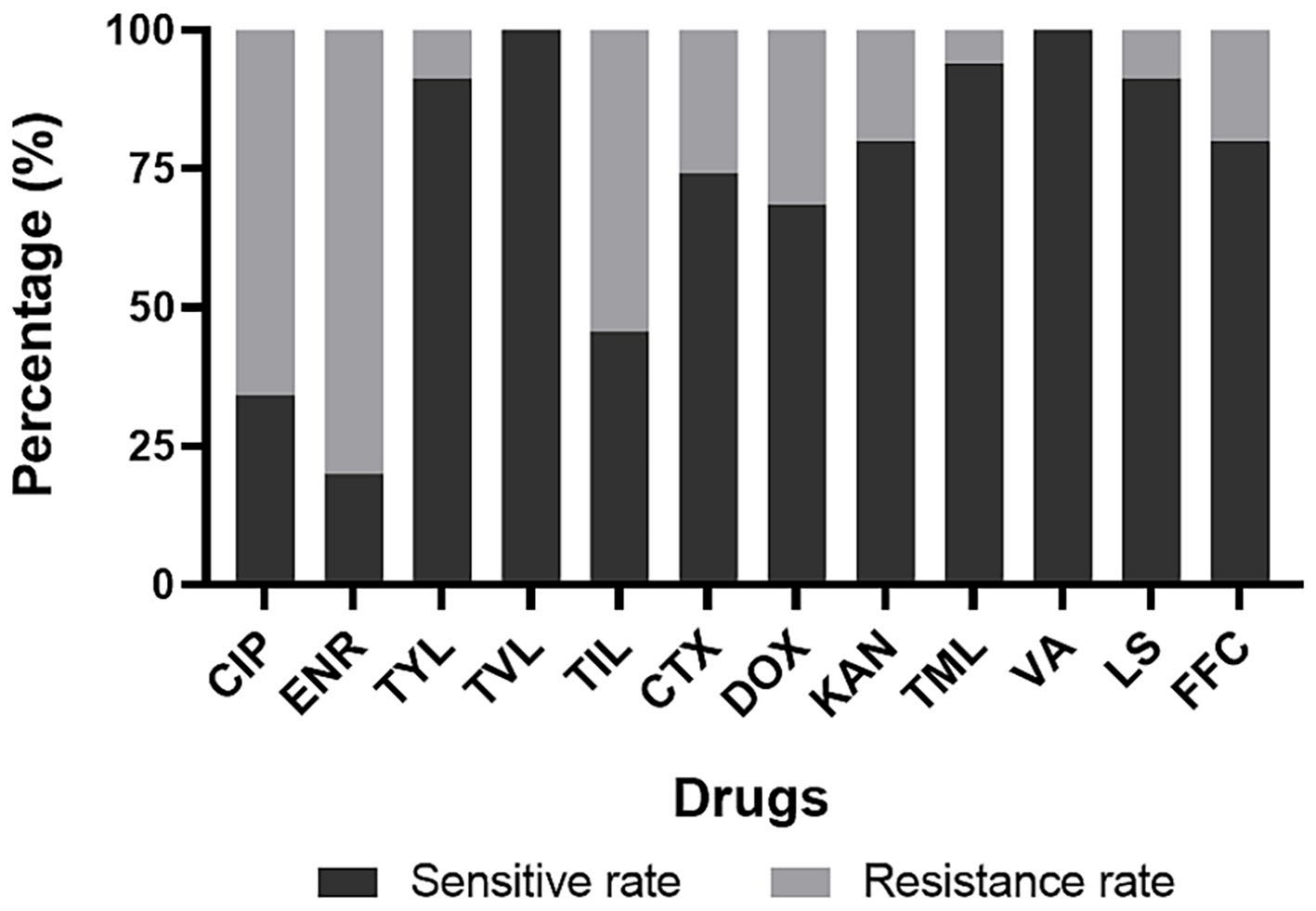
Resistance rates of MS isolates to 12 antimicrobial agents.

Based on established susceptibility breakpoints for MS, the highest resistance rate was observed for ENR (80.0%), followed by CIP (65.7%), TIL (54.3%), DOX (31.4%), CTC (25.7%), KAN (20.0%), FFN (20.0%), TYL (8.8%), LS (8.8%), and TIA (5.9%). No resistant strains were detected against TVL or VAL. Most isolates were resistant to three or four classes of antibiotics, and two strains exhibited resistance to all six classes tested (Figure 1).

### 3.2 Molecular characterization of quinolone resistance genes

To investigate the molecular mechanisms underlying multidrug resistance in MS, sequencing analysis of antimicrobial resistance genes was performed. Given the increasing severity of bacterial resistance to antimicrobial agents, the quinolone resistance-determining regions (QRDRs) and the macrolide-associated resistance region of the 23S rRNA gene were sequenced in thirty-five MS isolates. The resulting sequences were compared with those of reference strains to identify resistance-associated mutations.

The reference strain MS-H (MIC = 0.03 μg/mL) was more susceptible to ENR than ATCC 25204 (MIC = 0.5 μg/mL), whereas 35 field isolates exhibited higher MIC values ranging from 0.5 to 16 μg/mL. Multiple non-synonymous mutation sites were identified upon comparing the QRDRs between the reference strains and the MS isolates (Table 3). In the GyrA protein, MS-H encodes glutamate (Glu) at position 63 and threonine (Thr) at position 181, while all thirty-five field isolates carry glycine (Gly) at position 63 and alanine (Ala) at position 181. In the ParE protein, both reference strains exhibit proline (Pro) at position 446, whereas all field isolates harbor serine (Ser) at this site. Additionally, two field isolates display a valine- to-glycine (Val348Gly) substitution.

At amino acid position 85 of ParC, three isolates and both reference strains retained Thr, whereas the remaining thirty-two isolates carried an isoleucine (Ile) substitution. Among 6/35 MS isolates sensitive to ENR, both amino acids at positions 345 and 417 in the GyrB protein were asparagine (Asn), while in the other 29 strains with higher MIC values, substitutions of Asn345Ser and Asn417Ser occurred at these two sites (Table 3).

### 3.3 Molecular characterization of macrolide resistance genes

Two nucleotide positions in domain II of the 23S rRNA gene exhibited sequential variations associated with macrolide susceptibility. At position 788, the nucleotide sites of 4 MS isolates from earlier years (2018: 2 isolates, 2020: 1isolate and 2021 1 isolate) were G nucleotide and showed low MICs to tilmicosin and tylosin. All remaining 31 isolates carry the G788C substitution and exhibit markedly mutated to elevated MICs for several times. At nucleotide position 915, 8 isolates collected between 2018 and 2023 shared the same G as the reference strain, whereas the other 27 strains had mutated to C, which was also associated with increased MICs to TIL and TYL. At position in domain VI, thirty-one isolates exhibited a G-to-C mutation, and notably, all these mutant strains carried G788C. All isolates had A at positions 2053 and 2054 in domain V of the 23S rRNA gene (equivalent to *E. coli* positions 2058 and 2059), with no variation detected (Table 4).

**Table 4:** Analysis of nucleotide/amino acid sites associated with macrolide drug resistance genes.

Genes/protein		Positions SNP/AA	WVU1853/MS-H	Mutation	Number of strains	TIL MIC (μg/mL)	TYL MIC (μg/mL)	Year
23S rRNA	Domain II	788	G	G	4	0.125–0.25	0.0156–0.062	2018–2021
				A	31	0.25–8	0.031–4	2019–2025
		915	G	G	8	0.125–1	0.0156–0.125	2018–2023
				C	27	0.25–8	0.031–4	2019–2025
	Domain VI	2,761	G	G	4	≤0.25	0.0156–0.062	2018–2021
				C	31	0.25–8	0.031–4	2019–2025
	Domain V	2058,2059	A	A	35	0.125–8	0.0156–4	2018–2025
Ribosomal L4		66	S	G	35	0.125–8	0.0156–4	2018–2025
		159	N	S	35	0.125–8	0.0156–4	2018–2025
		215	N	S	35	0.125–8	0.0156–4	2018–2025
Ribosomal L22		90	Q	Q	33	0.125–8	0.0156–4	2018–2025
				H	2	0.25, 1	0.031, 0.25	2018、2024

Compared with the two reference strains, all thirty-five isolates carried three nonsynonymous mutations in the *rplD* gene (encoding ribosomal protein L4): A196G, A476G, and A644G, resulting in the amino acid substitutions Ser66Gly, Asn159Ser, and Asn215Ser, respectively. In the *rplV* gene (encoding ribosomal protein L22), two MS isolates had a glutamine (Gln) to histidine (His) mutation at position 90.

## 4 Discussion

As a major pathogen threatening poultry health, MS has recently become one of the three most prevalent bacterial infections. Because of the abuse of multiple antimicrobial drugs (including quinolones, macrolides, tetracyclines, pleuromutilins, and others), control efficacy of drugs against MS is declining (11–13). Although resistance mechanisms are well-characterized in other Mycoplasmal species, they remain unclear in MS. In this study, we assessed the MICs of 12 common antimicrobial agents against 35 MS isolates collected from 2018 to 2025 and investigated the genetic basis of their resistance profiles.

Extensive susceptibility testing has demonstrated that various bacteria, including *Escherichia coli*, *Salmonella*, and *Streptococcus*, have developed resistance to ENR and CIP, which are broad-spectrum, highly effective third-generation fluoroquinolone antimicrobials (14–16). In the present study, the MICs of CIP against MS isolates ranged from 0.25 to 32 μg/mL. Based on established susceptibility breakpoints, 23 of the 35 MS isolates were resistant, corresponding to a resistance rate of 65.7%. For ENR, MICs ranged from 0.5 to 16 μg/mL, with a resistance rate of 80%. These findings are consistent with those of most other studies, with the exception of a report on South African MS isolates, which were found to be susceptible to low concentrations of enrofloxacin (MIC range: 0.08–0.64 μg/mL) (5, 17).

The MICs of TVL against the 35 MS isolates ranged from 0.0039 to 0.5 μg/mL, while those of TYL ranged from 0.0156 to 4 μg/mL, indicating high susceptibility of these isolates to macrolides. These results are consistent with previous studies showing that isolates from China (2016–2019) and Central and Eastern Europe (2002–2016) exhibited similar susceptibility to macrolides (18). Pleuromutilins have demonstrated marked efficacy against avian mycoplasmas, and to date, no insensitive strains have been reported globally, with MICs ranging from 0.039 to 0.312 μg/mL in Asian countries and from 0.039 to 0.625 μg/mL in Central and Eastern Europe (10). Similar results were obtained in the present study: MICs of VAL were uniformly low (0.002–0.0156 μg/mL) across all 35 isolates, and 33 isolates exhibited low MICs to TIA, indicating that resistance of MS to pleuromutilins remains comparatively stable. Previous studies also reported low MICs of tetracyclines against MS isolates in Asia (including China and Thailand) and in Europe (including Hungary, Italy, the Netherlands, Israel, and Spain) (14, 19). However, in the present study, the MICs of CTC and DOX against the 35 MS isolates ranged from 0.031 to 16 μg/mL and from 0.062 to 16 μg/mL, respectively, with resistance rates of 25.7 and 31.4%, which are higher than previously reported. These results indicate that resistance of MS to tetracyclines has increased in recent years, and the use of these drugs should therefore be reduced.

Bacterial resistance is associated with DNA gyrase genes, including *gyrA*, *gyrB*, *parC*, and *parE*, as well as domain I to VI of the 23S rRNA gene (14, 15, 20, 21). In the present study, we analyzed the sequences of these genes to elucidate the resistance mechanisms of MS. Previous studies have demonstrated that amino acid substitutions in parC alter bacterial susceptibility to fluoroquinolones (22, 23). Our results also showed that a T85I substitution in parC (present in 32 isolates) reduced fluoroquinolone susceptibility, with most MIC values exceeding 2 μg/mL. A similar substitution was identified in gyrB: isolates harboring the N345S substitution exhibited reduced susceptibility to enrofloxacin (MICs ≥2 μg/mL), whereas isolates lacking this substitution were more susceptible (MICs ≤1 μg/mL). In addition to these resistance-altering substitutions, we also identified several mutations that had little impact on MS susceptibility. For example, all 35 isolates in our study contained Ser at position 446 of parE, whereas previously reported strains carried Pro at this position. Furthermore, 31 isolates shared concurrent nucleotide substitutions in domain II (G778A) and domain VI (G2761C) of the 23S rRNA gene, and 27 isolates carried an additional G915C substitution in domain II. Although previous studies have suggested that these mutations may be associated with reduced susceptibility to macrolides (24–27), further investigations are required to evaluate the functional roles of these substitutions.

Comprehensive time-series trend analysis reveals that, among the MS isolates monitored in this study, both fluoroquinolone and macrolide resistance phenotypes and genotypes exhibit temporal clustering. Early isolates collected in 2018 were predominantly wild-type, exhibiting high susceptibility to fluoroquinolones (no mutations in ParC) and macrolides (no mutations in 23S rRNA, ribosomal proteins L4 or L22), with ENR MICs ≤ 1 μg/mL and CIP MICs ≤ 0.25 μg/mL. A marked shift in resistance profiles was observed starting in 2019. Most of circulating strains acquired a specific dual mutation genotype (ParC N85I and GyrB N345S), leading to a dramatic surge in fluoroquinolone MICs (ENR: up to 16 μg/mL; CIP: up to 32 μg/mL). This timeline paralleled the emergence of high-level macrolide resistance, driven primarily by 23S rRNA mutations (G788A, G915C, and G2761C) and compensatory L4 protein mutations. These genetic changes conferred significantly elevated MICs against TYL and TIA, and the persistence of these genotypes through 2025 suggests the clonal expansion of multidrug-resistant lineages within the study region. Collectively, 2019 represented a critical inflection point characterized by the concurrent emergence of fluoroquinolone-resistant strains harboring ParC/GyrB dual mutations and macrolide-resistant strains carrying 23S rRNA mutations.

The *in vivo* efficacy of antimicrobials against MS depends not only by MIC values but also critically on pharmacokinetic–pharmacodynamic (PK–PD) properties, tissue distribution, and host immune status (28–30). Due to the absence of a cell wall and inherently limited metabolic capacity, mycoplasmas exhibit unique challenges for antimicrobial penetration and target-site binding efficiency are particularly critical determinants of therapeutic success. Therefore, relying solely on MIC data without contextual integration of PK–PD parameters and relevant clinical factors may result in suboptimal treatment decisions and an underestimation of antimicrobial resistance risk.

## 5 Conclusion

In conclusion, 35 MS isolates had low MICs for VAL and TYV, with values ranging from 0.002 to 0.5 μg/mL. However, most MS isolates showed intermediate MICs for DOX, CTC, TYL, TIL, KAN, TIA and LS. High MICs for ENR, CIP and FFC were detected in all isolates. Sequence analysis revealed two nonsynonymous mutations within the QRDR: Asn345Ser in GyrB (detected in 29/35 isolates) and Thr85Ile in ParC (32/35 isolates), previously related to ENR resistance. In the 23S rRNA gene, three mutations: G788A (31/35 isolates), G915C (27/35 isolates), and G2761C (31/35 isolates) were identified; these substitutions were initially reported in association with reduced susceptibility to macrolide antibiotics, particularly TIL and TYL.

## Data Availability

The original contributions presented in the study are included in the article/supplementary material, further inquiries can be directed to the corresponding author.
